# Towards HCV extinction with modern HCV treatment? “*Yes we can!*”

**DOI:** 10.1186/1471-2334-12-S2-S1

**Published:** 2012-11-12

**Authors:** C Torti, A Focà, G Carosi

**Affiliations:** 1 Unit of Infectious Diseases, University “Magna Graecia”, Catanzaro, Italy; 2 Unit of Microbiology, University “Magna Graecia”, Catanzaro, Italy; 3 Department of Infectious Diseases, University of Brescia, Brescia, Italy

## 

With the availability of new drugs (*Directly Acting Antivirals*, DAA), the ambition to eradicate HCV infection even in patients infected by “difficult-to-treat” genotypes is becoming a reality. Benefits are enormous from an individual and a global perspective since about 3% people in the world are chronically infected, with a burden of 350,000 deaths related to the long-term complications of the disease (cirrhosis and hepato-cellular carcinoma).

The lessons we learned from HIV treatment (*Highly Active Antiretroviral Therapy*, HAART) are that antivirals pay-off by saving costs of clinical complications, patients’ stress and sufferance, and an increase in productivity of the infected individuals can be achieved for the sake of the society. Moreover, control of HIV replication induced by HAART can reduce the infection burden in the general population by decreasing HIV transmissibility [1]. This is extremely important for infection control when coupled with behavioural interventions since for HIV (as for HCV) an effective vaccine is not yet available. So, with highly active anti-HCV treatments we can now follow in HAART’s footsteps and even get ahead of HAART cost-effectiveness because HCV is eradicable and treatment can be stopped; by contrast, HAART must be continued life-long with a consequent incremental cost.

However, the road is still long and we should overcome several obstacles along the way. First, benefits of HCV treatment are maximal if treatment is prescribed, ideally, to all patients in need. Unfortunately, HCV epidemic is largely underground as most HCV infected individuals are unaware of their infection status. It is clear that screening policy should be optimized to detect as many cases as possible but it is unclear what is the most cost-effective strategy. The article by Zaltron *et al.*, in this supplement discusses the epidemiological and clinical relevance of chronic HCV infection, including a review of the screening strategies and their cost-effectiveness [2]. The US Department of Health and Human Services has recently recommended HCV testing for “baby boomers” (i.e., adults born in the period 1945-65) as well as for adults of any ages reporting risk factors for HCV infection.

Second, in the current economic crisis, the paradox is that we should treat as many patients as possible but resources are limited in nature. Therefore, our focus must be on improving cost-effectiveness. With this objective in mind, three main questions for clinicians are: (i) *Whom to treat?* (ii) *How to treat?* (iii) *When to treat?* The expert-opinion paper by Petta & Craxì tries to answer to these questions [3]. For example, these authors underline that, by considering the initial virological response (*Rapid Virological Response*, RVR) and a new pharmacogenomics test (IL28B genotype) as predictors of treatment success, it is possible to limit the use of DAA with saving in cost and drug toxicity. Indeed, a standard treatment with Peg-interferon and ribavirin provides a similar rate of success as a “triple” regimen including a DAA in 25-33% of patients, provided that a prognostic score (including pharmacogenomics screening) is favourable and a RVR is achieved. In their papers, Nucara *et al.*, review the key data on the use of IL28B genotype in clinical practice [4], while Colucci describes the molecular tests available as diagnostic tools and surrogate markers of treatment response [5].

Third, a lesson we learned is the importance of treatment adherence and retention into care [6]. So, clinicians should provide the maximum support to improve patients’ adherence to treatment and increase the rate of success. A dedicated approach with a strict follow-up should be in place, not only dealing with adverse events and assessing the virological response for a rapid adaptation of treatment, but also providing a psychosocial support to patients in need. Only through an inter-disciplinary collaboration following a patient-centred approach –with clinicians (specialists in infectious diseases, gastroenterology, and hepathology), virologists, and psychologists working together - we can improve our standard of care.

Fourth, the holy grail of HCV eradication at the population level could be achieved only if HCV transmission is controlled both with treatment and behavioural intervention. Along this line, it is important to define HCV transmission routes, identify the most-at-risk population and implement targeted prevention strategies. As appropriately underlined in the papers by Liberto *et al., *[7] and by Ciccozzi *et al., *[8], monitoring the genetic evolution of HCV is an essential step to develop efficient preventative measures for controlling HCV epidemic. Also, epidemiologic estimates and HCV genotyping will help public health officials to allocate appropriate health-care resources for managing this important condition.

What is the way forward? New weapons against HCV are in the pipeline, allowing the construction of combination regimens taken orally. Phase 2 trials conducted in patients naïve to treatment support the efficacy of these convenient regimens without the addition of Peg-interferon and ribavirin. In their paper, Puoti *et al.*, conclude that the advent of these regimens is eagerly awaited but caveats in the results of phase 2 trials suggest to dampen enthusiasm and conduct more studies on how to better use these innovative drugs [9].

Against this background we feel that it is important to set-up collaborative networks to implement operational research. The SINERGIE (*South Italian Network for Rational Guidelines and International Epidemiology*) project –object of the last article of the supplement [10]– will focus on upcoming research questions, with the final aim of improving the identification, care of patients, prevention strategies and health resource allocation in the Calabria Region (Southern Italy). This is a “global”, patient-centred research. We hope that this project will be extended to other Regions. Indeed, it is now time for a coordinated, inter-disciplinary effort to better use the new weapons that we have and those available in the future. Only with a better knowledge and understanding we can improve our skills and ability to fight an insidious enemy such as HCV, driving it to extinction.

## Competing interests

The authors declare that they have no competing interests related to the contents of this paper.

## Declarations

Publication of this supplement was partly supported by an unrestricted grant provided by Roche. The articles were independently prepared by the authors with no input from Roche. Roche were not involved in selecting the articles for the supplement. The Peg-interferon treatment mentioned in this article is produced by Roche. 

## References

[B1] Cohen MS Chen YQ McCauley M Prevention of HIV-1 infection with early antiretroviral therapy New England Journal of Medicine 2011 365 493 505 10.1056/NEJMoa1105243 21767103 PMC3200068

[B2] Zaltron S Spinetti A Biasi L Baiguera C Castelli F Chronic HCV infection: epidemiological and clinical relevance BMC Infectious Diseases 2012 12 Suppl 2 S2 10.1186/1471-2334-12-S2-S2 PMC3495628 23173556

[B3] Petta S Craxì A Therapeutic algorithms for chronic hepatitis C in the DAA era during the current economic crisis: whom to treat? How to treat? When to treat? BMC Infectious Diseases 2012 12 Suppl 2 S3 10.1186/1471-2334-12-S2-S3 PMC3495629 23173606

[B4] Nucara S Caroleo B Guadagnino V Perrotti N Trapasso F Natural history and clinical response: “It’s the virus, stupid, or is it the host?” BMC Infectious Diseases 2012 12 Suppl 2 S6 10.1186/1471-2334-12-S2-S6 PMC3495625 23173731

[B5] Colucci G Molecular diagnostic and predictive tests in the evolution of chronic hepatitis C anti-viral therapies BMC Infectious Diseases 2012 12 Suppl 2 S8 10.1186/1471-2334-12-S2-S8 PMC3495636 23173776

[B6] Lo Re V Amorosa VK Localio AR Adherence to hepatitis C virus therapy and early virologic outcomes Clinical Infectious Diseases 2009 48 186 193 10.1086/595685 19086908 PMC2668718

[B7] Liberto MC Marascio N Zicca E Matera G Epidemiological features and specificities of HCV infection: a hospital-based cohort study in a University Medical Center of Calabria region BMC Infectious Diseases 2012 12 Suppl 2 S4 10.1186/1471-2334-12-S2-S4 PMC3495624 23173638

[B8] Ciccozzi M Lo Presti A Ciccaglione AR Zehender G Ciotti M Phylogeny and phylodinamic of Hepatitis C in Italy BMC Infectious Diseases 2012 12 Suppl 2 S5 10.1186/1471-2334-12-S2-S5 PMC3495630 23173700

[B9] Puoti M Rossotti R Travi G Orso M Moioli MC Top topics in HCV research arena BMC Infectious Diseases 2012 12 Suppl 2 S7 10.1186/1471-2334-12-S2-S7 PMC3495634 23173752

[B10] Torti C Zazzi M Abenavoli L Trapasso F Cesario F Corigliano M Cosco L Costa C Curia RL De Rosa M Foti G Giraldi C Leone R Liberto MC Lucchino D Marascio N Masciari R Matera G Pisani V Serrao N Surace L Zicca E Castelli F Ciccozzi M Puoti M Focà A SINERGIE Study Group Future research and collaboration: the “SINERGIE” project on HCV BMC Infectious Diseases 2012 12 Suppl 2 S9 10.1186/1471-2334-12-S2-S9 PMC3495626 23173812

